# Transcriptional profiles of *Burkholderia pseudomallei* reveal the direct and indirect roles of Sigma E under oxidative stress conditions

**DOI:** 10.1186/1471-2164-15-787

**Published:** 2014-09-12

**Authors:** Siroj Jitprasutwit, Catherine Ong, Niramol Juntawieng, Wen Fong Ooi, Claudia M Hemsley, Paiboon Vattanaviboon, Richard W Titball, Patrick Tan, Sunee Korbsrisate

**Affiliations:** Department of Immunology, Faculty of Medicine Siriraj Hospital, Mahidol University, Bangkok, 10700 Thailand; DSO National Laboratories, Singapore, 117510 Singapore; Genome Institute of Singapore, Singapore, 138672 Singapore; Bioscience, Geoffrey Pope Building, University of Exeter, Devon, EX4 4QD UK; Laboratory of Biotechnology, Chulabhorn Research Institute, Bangkok, 10210 Thailand

**Keywords:** *B. pseudomallei*, Transcription profile, Sigma E, SpeG, Oxidative stress

## Abstract

**Background:**

*Burkholderia pseudomallei*, the causative agent of melioidosis, is a Gram-negative bacterium widely distributed in soil and water in endemic areas. This soil saprophyte can survive harsh environmental conditions, even in soils where herbicides (containing superoxide generators) are abundant. Sigma factor E (σ^E^) is a key regulator of extra-cytoplasmic stress response in Gram-negative bacteria. In this study, we identified the *B. pseudomallei* σ^E^ regulon and characterized the indirect role that σ^E^ plays in the regulation of spermidine, contributing to the successful survival of *B. pseudomallei* in stressful environments.

**Results:**

Changes in the global transcriptional profiles of *B. pseudomallei* wild type and σ^E^ mutant under physiological and oxidative stress (hydrogen peroxide) conditions were determined. We identified 307 up-regulated genes under oxidative stress condition. Comparison of the transcriptional profiles of *B. pseudomallei* wild type and σ^E^ mutant under control or oxidative stress conditions identified 85 oxidative-responsive genes regulated by σ^E^, including genes involved in cell membrane repair, maintenance of protein folding and oxidative stress response and potential virulence factors such as a type VI secretion system (T6SS). Importantly, we identified that the *speG* gene, encoding spermidine-acetyltransferase, is a novel member of the *B. pseudomallei* σ^E^ regulon. The expression of *speG* was regulated by σ^E^, implying that σ^E^ plays an indirect role in the regulation of physiological level of spermidine to protect the bacteria during oxidative stress.

**Conclusion:**

This study identified *B. pseudomallei* genes directly regulated by σ^E^ in response to oxidative stress and revealed the indirect role of σ^E^ in the regulation of the polyamine spermidine (via regulation of *speG*) for bacterial cell protection during oxidative stress. This study provides new insights into the regulatory mechanisms by which σ^E^ contributes to the survival of *B. pseudomallei* under stressful conditions.

**Electronic supplementary material:**

The online version of this article (doi:10.1186/1471-2164-15-787) contains supplementary material, which is available to authorized users.

## Background

*Burkholderia pseudomallei* is a Gram-negative bacterium and the causative agent of melioidosis [1, 2]. This serious and often fatal disease of humans and animals such as horses, sheep, goats, pigs and cows is endemic in Southeast Asia and northern Australia [1, 3]. *B. pseudomallei* is intrinsically resistant to several antibiotics and treatment typically involves an initial parenteral phase of therapy, followed by a prolonged course of oral antibiotics [4]. No melioidosis vaccine is currently available. In endemic areas, *B. pseudomallei* can be found in soil and in stagnant waters [5]. In the natural environment, this saprophytic bacterium is able to survive a wide range of conditions, including fluctuating temperatures, pH levels, oxygen levels, osmotic pressures and nutritional stresses. During infection, the bacterium is able to survive and replicate in phagocytic or non-phagocytic cells. Within these cells *B. pseudomallei* may be exposed to free radicals, reactive oxygen intermediates and high osmolality. To survive exposure to stressful environments, *B. pseudomallei* must be able to activate the appropriate genes and regulate their expression. Many of these genes are organized into regulons which are under the control of sigma factors.

RpoE (σ^E^) is a member of the extra-cytoplasmic function (ECF) subfamily of sigma factors [6] and has been characterized to be one of the most important gene regulatory systems in response to extracellular stress in Gram-negative bacteria. In *Escherichia coli* K12, the inhibition of σ^E^ resulted in increased sensitivity to bacterial cell wall disruption [7] and in *Vibrio vulnificus*, deletion of σ^E^ resulted in increased sensitivity to membrane-perturbing agents such as ethanol, peroxide and SDS [8]. Inactivation of σ^E^ in *Salmonella enterica* serovar Typhimurium (*S.* Typhimurium) resulted in attenuation in a mouse model of infection [9, 10]. In addition, microarray analysis of a *S.* Typhimurium σ^E^ mutant identified the σ^E^ regulon and virulence factors that contributed to disease [11, 12].

A *B. pseudomallei rpoE* insertional inactivation mutant has previously been constructed and showed increased susceptibility to hydrogen peroxide (H_2_O_2_), suggesting a role for σ^E^ in the oxidative stress response [13]. Furthermore, inactivation of *B. pseudomallei* σ^E^ resulted in reduced survival in J774A.1 macrophages and the mutant was attenuated in a murine model of infection [13, 14]. A proteomic comparison of *B. pseudomallei* wild type and the σ^E^ insertional mutant revealed the differential levels of proteins that may contribute to the stress tolerance and survival of *B. pseudomallei* [14] but this study was unable to identify all the proteins involved in this response, because of the limitations of the proteomic platform. Recently, the development of a tiling microarray for *B. pseudomallei* enabled comprehensive transcriptional profiling, providing global snapshots of regulons in response to various stimuli. Analyses of transcriptional profiles of σ^E^ would lead to a better understanding of the mechanisms that bacteria use to circumvent environmental stresses. Such microarray studies will also complement our previous proteomic data and is likely to provide new insights to gene members and regulation of these genes under stress.

In this study, global transcriptional profiles of *B. pseudomallei* in response to H_2_O_2_-induced oxidative stress were analyzed. We compared the transcriptional profiles of *B. pseudomallei* wild type and its isogenic σ^E^ mutant under oxidative stress. In addition, the transcriptional profiles also revealed a novel gene member of the σ^E^ regulon, *speG*, that is involved in maintaining the physiological balance of the polyamine spermidine in bacterial cells during oxidative stress. This is the first report to demonstrate the direct and indirect roles of σ^E^ contributing to *B. pseudomallei* survival in the environment.

## Results and discussion

### Comparative transcriptional profiles of *B. pseudomallei*wild type with and without oxidative stress

**Figure 1:**
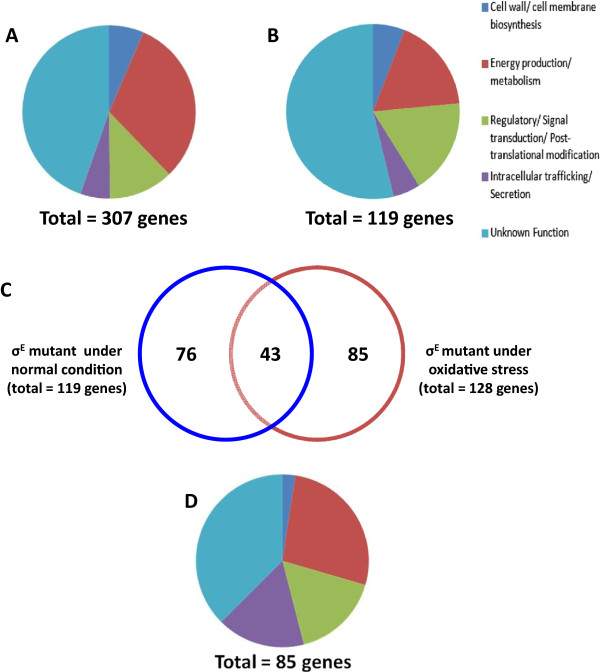
**Differentially expressed genes. A)** Up-regulated genes under oxidative stress in wild type *B. pseudomallei* K96243. **B)** Down-regulated genes under control condition (LB broth) in the σ^E^ mutant. **C)** Comparison of microarray profiles from σ^E^ mutant under control and oxidative stress conditions shows 85 genes as σ^E^-dependent oxidative-stress-responsive genes. **D)** Functional classification of the 85 σ^E^-dependent oxidative-stress-responsive genes.

The transcriptional profiles of *B. pseudomallei* in the presence or absence of 100 μM H_2_O_2_ for 10 min were first determined. Analyses of the profiles revealed a total of 649 genes (Additional file 1) that were differentially regulated (≥1 absolute log-transformed fold change) representing approximately 11.0% of all *B. pseudomallei* K96243 genes. These differentially regulated genes were found on chromosome 1 (57.5%) and chromosome 2 (42.5%). Among the 649 genes, 307 genes were up regulated (47.3% of differentially regulated genes) and 342 genes (52.7%) were down regulated under oxidative stress. Since the objective of this study was to identify *B. pseudomallei* gene expression in response to oxidative stress, we focused on the analysis of the up-regulated genes. Among 307 up-regulated genes, 221 (72.0%) could be classified into 4 major functional groups according to the Cluster of Orthologous Groups of proteins (COGs) database. These included genes involved in cell wall/membrane biosynthesis; energy and metabolism; regulatory, signal transduction and post-translational modification; intracellular trafficking/secretion system. The remaining 86 genes (28.0%) had unknown functions (Figure 1A).

Of the 307 up-regulated genes, 20 genes (6.5%) were involved in cell wall/cell membrane biosynthesis (Figure 1A) including *bpsl0497* (encoding periplasmic TonB protein)*, bpsl0785* (encoding a peptidase), *bpsl3312* (encoding a putative glycosyltransferase), *bpss0238* (encoding a penicillin-binding protein) and *bpss0711* (encoding an alanine racemase). We also found that genes involved in the transport of lipopolysaccharide (*bpsl0963*) and capsular polysaccharide *(bpsl*2806) across the cell membrane to the bacterial cell surface were up-regulated. We found increased expression of *mreB* (*bpsl0186*) gene after oxidative stress. MreB is a bacterial ortholog of actin and MreB is reported to be important in maintaining the shape of bacteria [15, 16]. MreB is thought to organize the incorporation of cell wall precursors into the side-wall [17]. The up-regulation of genes involved in cell wall/cell membrane synthesis may reflect their roles in the repair of the cell wall after oxidative stress damage.

In addition to genes involved in cell wall and membrane biosynthesis, 87 genes involved in energy production and metabolism were up-regulated (31% of all up-regulated genes). These included three genes belonging to the sugar transporter superfamily (*bpsl1045, bpsl2729*, and *bpsl2736*), and two genes of the Entner-Doudoroff pathway (*bpsl2931* and *bpsl2932*). In addition, many genes related to amino acid utilization (*bpsl1076, bpsl2305* and *bpsl2497*) and amino acid biosynthesis (*bpsl3419*) were up-regulated when *B. pseudomallei* was exposed to oxidative stress. We found an increased expression of *B. pseudomallei bpsl1784* (encoding ATP-binding cassette transporters) which plays a role in inorganic iron transport. A previous study [18] reported that the bioavailability of iron decreases under oxidative stress conditions, and the up-regulation of *bpsl1784* is consistent with this observation. The increased expression of *B. pseudomallei fis*-regulatory gene (*bpsl0609*) suggests that BPSL0609 interacts with σ^54^ (a nitrogen specific sigma factor), with the consequential transcription of genes involved in the metabolism and transportation of nitrogen and carbon and genes involved in alginate and flagella synthesis [19, 20].

We observed an increase in the expression of peroxide scavenging enzymes including *katG* (catalase-peroxidase; *bpsl*2865), *katB* (monofunctional catalase; *bpss0993*) and *ahpC* (alkyl hydroperoxide reductase; *bpss*0492) during exposure to H_2_O_2_-induced oxidative stress (Table 1). The expression of *B. pseudomallei katG* and *ahpC* is regulated through a global H_2_O_2_ sensor and the OxyR transcriptional regulator [21–23]. The increased expression of *katG* and *ahpC* after exposure to oxidative stress is consistent with the findings from previous studies [22, 23]. The role of KatG may be to enable *B. pseudomallei* survival within phagocytes through the detoxification of antibacterial reactive oxygen species.

**Table 1:** **Selected differentially regulated genes of** ***B. pseudomallei*** **K96243 and σ** ^**E**^ **mutant under H**_**2**_ **O**_**2**_ **–induced oxidative stress**

Gene loci	Description of gene product	Fold change: wild type under oxidative stress compared with untreated control ^*^	Fold change: σ ^E^ mutant compared with wild type under oxidative stress
**Oxidative stress responsive gene (OSR gene)**			
*bpss0993*	KatB	5.90	NS
*bpss0492*	AhpC	4.54	NS
*bpsl2865*	KatG	3.64	NS
*bpss0238*	Penicillin-binding protein	3.03	NS
*bpss0281*	4 Aminobutyrate aminotransferase	2.80	NS
*bpss0711*	Alanine racemase	2.25	NS
*bpsl3142*	BolA-like protein	1.94	NS
*bpsl2285*	Chaperone protein HscA	1.88	NS
*bpsl0497*	Periplasmic TonB protein	1.54	NS
*bpsl2286*	Co-chaperone HscB	1.43	NS
*bpsl1787*	ECF sigma factors	1.41	NS
*bpsl0785*	Peptidase	1.19	NS
*bpsl0963*	Putative permease protein	1.13	NS
*bpsl2806*	Capsular polysaccharide	1.07	NS
*bpss0585*	AraC family transcriptional regulator	1.07	NS
*bpsl1527*	Tex transcriptional factor	1.05	NS
*bpsl0186*	MreB	1.04	NS
*bpsl3312*	Putative glycosyltransferase	1.00	NS
**σ** ^**E**^ **-dependent and OSR gene**			
*bpss1837*	Hypothetical protein	3.37	−1.84
*bpss1839*	Oxidative stress related rubrerythrin protein	3.36	−1.68
*bpss1838*	Ferredoxin	3.31	−1.79
*bpsl2931*	Eda (KHG/KDPG_aldolase)	3.26	−2.59
*bpsl2932*	Phosphogluconate dehydratase	3.17	−2.13
*bpsl2605*	TrxB	2.75	−1.18
*bpsl1799*	Putative fimbrial chaperone	2.70	−1.71
*bpsl1800*	Putative outer membrane usher protein precursor	2.59	−1.86
*bpss1437*	Lipoprotein	2.42	−2.27
*bpss1251*	N-carbamoyl-L-amino N-acid amidohydrolase	2.34	−1.01
*bpss1434*	Membrane-anchored cell surface protein	2.33	−3.52
*bpsl3419*	Putative GMC oxidoreductase	2.27	−1.22
*bpss1252*	Inner membrane transport protein	2.20	−1.11
*bpsl2300*	PdhB	2.19	−1.34
*bpsl2301*	PdhA	1.89	−1.68
*bpss0175-0184*	T6SS-4		
	BPSS0175	1.89	−3.67
	BPSS0176	1.77	−3.55
	BPSS 0177	1.70	−3.78
	BPSS 0178	1.99	−2.92
	BPSS 0179	1.94	−2.56
	BPSS 0180	1.70	−2.50
	BPSS 0181	1.68	−2.16
	BPSS0182	1.14	−1.50
	BPSS0183	1.06	−1.09
	BPSS0184	1.04	−1.22
*bpsl2933*	Putative regulatory protein	1.61	−1.29
*bpsl1042*	Putative lipoprotein	1.61	−1.43
*bpsl1806*	Subfamily M23B unassigned peptidase	1.57	−1.60
*bpsl1983*	Putative two component system histidine kinase	1.41	−1.00
*bpss0796A*	H-NS-like protein	1.41	−1.32
*bpsl0609*	Fis family regulatory protein	1.31	−3.20
*bpsl0320*	PfkB family carbohydrate kinase	1.31	−1.10
*bpss0016*	Phospholipase	1.23	−2.72
*bpss0124*	Response regulator	1.22	−2.64
*bpsl0785*	Putative lipoprotein	1.19	−1.21
*bpss1133*	FadH	1.15	−1.30
*bpss2053*	Cell surface protein	1.12	−2.46
*bpsl1043*	Putative lipoprotein	1.12	−1.44
*bpsl3216*	FusA elongation factor EF-2	1.10	−1.10
*bpsl1577*	TkrA 2-ketogluconate reductase	1.06	−1.30
*bpsl1893*	Putative type II/IV secretion system ATP-binding protein	1.02	−1.01
**σ** ^**E**^ **-dependent but not OSR gene**			
*bpsl0096*	SpeG spermidine n(1)-acetyltransferase	NS	−1.29
*bpsl0224*	Putative GMC oxidoreductase	NS	−1.24
*bpsl0327*	LysR family regulatory protein	NS	−1.85
*bpsl2289*	IscS cysteine desulfurase	NS	−1.08
*bpss1944*	AdhA alcohol dehydrogenase	NS	−1.01
*bpss1945*	AtpG ATP synthase gamma chain	NS	−1.23
*bpss1946*	AtpA ATP synthase subunit A	NS	−1.24

NS; Not significant different.

*B. pseudomallei cultured in LB broth without H_2_O_2_.

The differential transcription profile of *B. pseudomallei* under oxidative stress revealed that 37 genes (12.1% of oxidative stress responsive genes) were predicted to encode regulatory, signal transduction or post-translational modification-related proteins (Figure 1A). These genes included *bpsl0049*, encoding a GntR family regulatory protein and *bpsl1787*, encoding an ECF sigma factor. Several genes involved in transcription regulation such as *tex* (*bpsl1527* encoding a transcriptional factor), *nrdR* (*bpsl2757* encoding a transcriptional regulator) and an *araC* family gene (*bpss0585* encoding a transcriptional regulator) were up-regulated. In *Streptococcus pneumoniae* and *Pseudomonas aeruginosa* the transcription factor Tex is important for bacterial fitness [24]. The NrdR transcription regulator is reported to control the expression of a ribonucleotide reductase involved in deoxyribonucleotide biosynthesis, which is required for DNA replication and repair [25]. Many members of the AraC family transcription regulator have been proven to play critical roles in regulating bacterial virulence factors in response to environmental stress [26]. The high number of regulatory genes up-regulated after exposure to oxidative stress may indicate that *B. pseudomallei* employs multiple regulation systems in response to oxidative stress.

In addition to genes involved in transcription, a number of chaperone-encoding genes were up-regulated after expsore of the bacteria to oxidative stress including *hscA/hscB* (*bpsl2285/bpsl2286*), and *groES*2 (*bpsl2919*). HscA is a specialized member of the *hsp70* family of molecular chaperones that plays a role in the biosynthesis of several iron-sulfur proteins [27]. Previous studies indicated the essential roles of iron-sulfur proteins in the adaptation of bacteria to iron starvation [28]. Chaperonin GroES2 binds to heat shock protein GroEL to facilitate protein folding in response to environmental stresses [29]. Oxidative stress can cause to protein misfolding, and as a result, the bacterial cells are unable to maintain their protein functions. The up-regulation of genes involved in protein folding may reflect the fact that under oxidative stress conditions, *B. pseudomallei* proteins are likely to become damaged.

The smallest functional group of proteins that were up-regulated under oxidative stress included 18 genes (5.5% of total up-regulated genes) encoding proteins related to intracellular trafficking and secretion (Figure 1A). Increased expression of proteins in this group, such as type II/IV and VI secretion systems implies that the virulence of *B. pseudomallei* is likely to be affected by oxidative stress.

### Comparative analysis of transcription profiles of *B. pseudomallei*wild type and σ^E^mutant without oxidative stress

We have previously reported the construction of *B. pseudomallei* σ^E^ mutant. The mutant shows increased susceptibility to killing by H_2_O_2_, indicating the role of σ^E^ in regulating resistance to oxidative stress [13, 14]. To identify the σ^E^ regulon under oxidative stress conditions, we first investigated the transcriptional profiles of *B. pseudomallei* wild type and the σ^E^ mutant grown in LB medium without antibiotic supplementation. Analysis of the transcription profiles revealed that a total of 350 genes (Additional file 2) were differentially regulated (≥1 absolute log fold change), representing approximately 5.9% of the total *B. pseudomallei* K96243 genes. These differentially regulated genes were distributed on both chromosome 1 (59.4%) and chromosome 2 (40.6%). In total, 231 genes were up-regulated (66.0% of differentially regulated genes) and 119 genes (34.0%) were down-regulated in the σ^E^ mutant. The down-regulation of genes may indicate either direct or indirect regulation by σ^E^. Among the down-regulated genes, 55 (46.2%) could be classified into 4 major COG functional groups, including 7 genes (5.9%) predicted to be involved in cell wall/cell membrane biosynthesis, 21 genes (17.6%) involved in energy production/metabolisms, 21 genes (17.6%) involved in regulatory/signal transduction/post-translational modification and repair, and 6 genes (5.1%) involved in intracellular trafficking/secretion (Figure 1B). The remaining 64 genes (53.8%) have unknown functions.

### Comparative analysis of transcription profiles of *B. pseudomallei*wild type and σ^E^mutant under oxidative stress

To identify σ^E^-dependent genes that are differentially expressed under oxidative stress conditions, we compared the transcriptome profiles of the σ^E^ mutant and wild type which had been exposed to oxidative stress. The bacteria were treated with H_2_O_2_ for 10 min before RNA extraction and microarray analysis. A total of 404 genes (Additional file 3) were differentially regulated (≥1 absolute log fold change) representing approximately 6.81% of the total *B. pseudomallei* K96243 genes. Of these, 276 genes were up-regulated in the σ^E^ mutant (68.3% of the total differentially regulated genes) and were located on either chromosome 1 (56.5%) or chromosome 2 (43.5%). Among the 128 down-regulated genes in the σ^E^ mutant, 43 genes were also down-regulated in the mutant under normal growth conditions. By excluding these genes, we identified 85 genes defined as the σ^E^-dependent oxidative stress regulon (Figure 1C). These 85 genes were distributed on both chromosome 1 (53.1%) and chromosome 2 (46.9%). Two genes (2.4%) were predicted to be involved in cell wall/cell membrane biosynthesis, 23 genes (27.1%) in energy production/metabolisms, 14 genes (16.5%) in regulatory/signal transduction/post-translational modification and repair, 14 genes (16.5%) in intracellular trafficking/secretion. The remaining 32 genes (37.5%) had unknown functions (Figure 1D).

Amongst the 85 genes making up the σ^E^-dependent oxidative stress regulon, *bpsl1806* is predicted to be involved in cell wall/cell membrane biosynthesis and *bpss0265* is predicted to encode a membrane protein related to metalloendopeptidases and porins. Genes involved in energy production and metabolism included *bpsl0320*-*0321* (sugar kinase and N-acyl-D-glucosamine 2-epimerase), *bpsl2931* (KHG/KDPG aldolase) and *bpsl2300*-*l2301* (pyruvate dehydrogenase complex). The absence of a functional σ^E^ under oxidative stress affected the expression of *bpss1838-1839* (encoding ferredoxin and rubrerythrin proteins), genes that play important roles in increasing tolerance and resistance to oxidative stress [30].

We identified 14 (16.5%) σ^E^-regulated genes involved in regulatory, signal transduction and post-translational modification after oxidative stress including *bpsl0609* (encoding fis-regulatory protein)*, bpsl1983* (putative two-component system, histidine kinase), *bpsl2933* (putative regulatory protein), *bpss0124* (two-component system, response regulator) and *bpsl2605* (*trxB*). The latter encodes thioredoxin reductase which functions in post-translational modification. In addition, site-specific recombinase (*bpsl2881*), which is involved in DNA replication, recombination and repair, was also under σ^E^ regulation.

Intracellular trafficking and secretion genes accounted for 12.8% of σ^E^-dependent oxidative stress responsive genes. These included genes of the type II secretion system (*bpsl1893*), fimbrial proteins (*bpsl1798-1800*) and membrane-anchored cell surface protein (*bpss1434*). *B. pseudomallei* contains six clusters type VI secretion system (T6SS-1 to T6SS-6) [31]. The expression of T6SS genes has been reported to be induced *in vivo* [31–33]. A previous study reported that the T6SS-1 cluster is important for host adaptation of *B. pseudomallei* within phagocytes, and that the expression of genes in this cluster is significantly elevated after infection of murine macrophages [31]. We found the increased expression of ten genes (*bpss0175-0184*) belonging to T6SS-4 under oxidative stress conditions suggesting that T6SS-4 may play a role in combating oxidative stress*.*

### RT-PCR analysis of genes under normal and oxidative stress conditions

**Figure 2:**
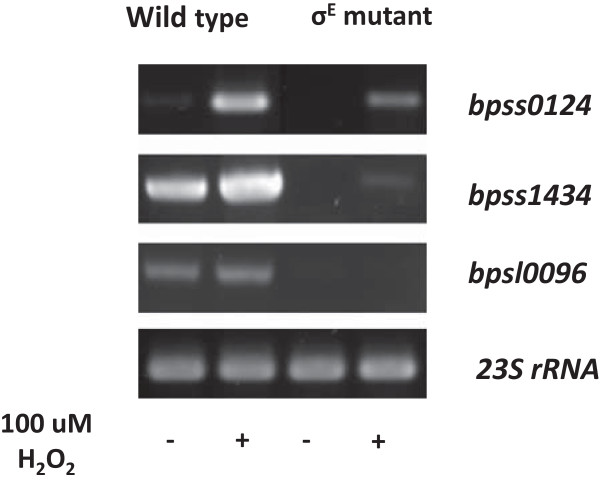
**RT-PCR analysis of genes under normal and oxidative stress conditions.** *B. pseudomallei* wild type or the isogenic σ^E^ mutant was incubated for 10 min in the presence or absence of 100 μM H_2_O_2_ and RT-PCR analysis carried out. Each row represents an individual gene (*bpsl0124*, *bpss1434* or *bpsl0096*) and normalized against 23S rRNA expression.

To validate the results from our microarray analysis, RT-PCR was performed. Figure 2 shows the increased expression of the *bpsl0124* and *bpss1434* genes in *B. pseudomallei* wild type after H_2_O_2_ treatment. We did not observe a significant difference in expression of *bpsl0096* (*speG*; encoding spermidine-n-1-acetyltransferase). These results are consistent with the microarray data which indicated that *bpsl0124* and *bpss1434*, but not *bpsl0096*, were up-regulated in response to oxidative stress (Additional file 1). In the *B. pseudomallei* σ^E^ mutant exposed to H_2_O_2_ treatment, the expression of the *bpsl0124*, *bpss1434* and *bpsl0096* genes was down-regulated compared to the wild type, indicating that these genes are under σ^E^ control. These results are also consistent with our microarray results (Additional file 2).

In addition to RT-PCR, the differential transcription profiles were analyzed to ensure the quality of our transcription profile data. For example, we found that 10 genes (*bpss0175-0184*) encoding *B. pseudomallei* T6SS-4 were all up-regulated, suggesting that these genes may be co-expressed as an operon and support the validity of our transcriptional profiling results (Table 1).

Other studies have shown that the *katG* (catalase-peroxidase) and *ahpC* (alkyl hyperoxide reductase) genes are up-regulated following the exposure of *B. pseudomallei* to oxidative stress [22, 23]. Our transcriptional data (Table 1) also reveals these patterns of gene expression. Collectively, these results indicate that our data is robust and reliable.

### *B. pseudomallei*σ^E^indirectly regulates spermidine levels during oxidative stress

Previous studies have suggested that sigma factors regulate *speG* and consequently spermidine levels [34, 35]. Spermidine is one of the predominant polyamines in Gram-negative bacteria, widely distributed in the environment, and is involved in various biological processes including gene regulation, protein translation and stress resistance [36]. During oxidative stress, spermidine functions as a free radical scavenger and plays an important adjunctive role in protecting bacterial cells from the toxic effects of reactive oxygen species [37]. The intracellular level of spermidine in bacteria is reported to range from 1-3 mM [38]. High concentrations of spermidine are toxic for bacteria. Excess spermidine can be a result of de-regulated bacterial biosynthesis/metabolism or from environmental exposure, inhibiting bacterial growth and even killing the bacterial cells [39]. Therefore, in bacteria, the maintenance of an appropriate intracellular level of spermidine is critical. Excess spermidine can be converted into the physiologically inert acetylspermidine by the spermidine-acetyltransferase (SpeG). A recent study revealed that the *speG* gene has been silenced by convergent evolution in *Shigella* and this resulted in elevated levesl of intracellular spermidine. As a result, the survival of *Shigella* under oxidative stress is enhanced, contributing to its successful pathogenic lifestyle [40].

We observed expression of *speG* (*bpsl0096*) in *B. pseudomallei* wild type under both control and oxidative stress conditions (Figure 2). However, the *speG* gene was down-regulated in the σ^E^ mutant (Additional file 3), indicating that the inactivation of *rpoE* effected the expression of *speG*. This suggests that *speG* is regulated by σ^E^. The decreased gene expression we have observed corroborates our previous proteomic study [14]. To our knowledge, this is the first report on the regulation of *speG* by σ^E^.

**Figure 3:**
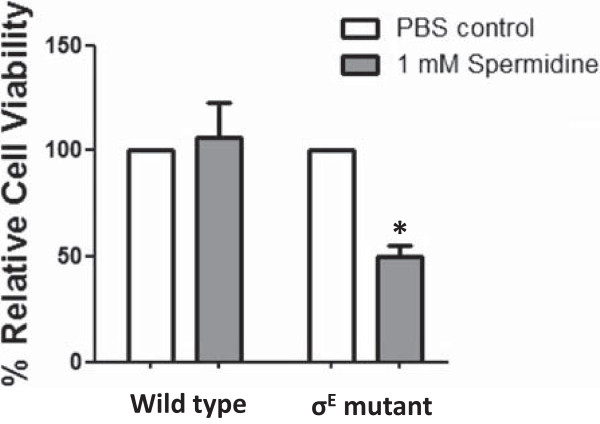
**Effect of spermidine on the viability of** ***B. pseudomallei*** **wild type or σ** ^**E**^ **mutant.** The numbers of *B. pseudomallei* wild type or the σ^E^ mutant cells were grown in the presence or absence of 1 mM spermidine. After 6 h, the numbers of viable bacteria (colony-forming unit; CFU) were determined after plating onto LB agar. The viability of wild type and σ^E^ mutant in the presence of spermidine was calculated from CFU count divided by the CFU count of control condition and multiplied by 100. Values shown are the mean of three independent experiments. Asterisks indicate significant differences (*P* = 0.038).

We hypothesised that under high concentrations of spermidine, σ^E^ positively regulate expression of s*peG* gene in order to prevent spermidine accumulation, the failure of which, will result in inhibition of *B. pseudomallei* growth and even cytotoxicity. To test this hypothesis, cultures of *B. pseudomallei* wild type or σ^E^ mutant grown in LB broth were exposed to 1 mM spermidine and the number of viable bacteria determined. The number of wild type bacteria was not affected by the addition of spermidine. This is likely due to the presence of a functional σ^E^ gene in the wild type, activating the expression of s*peG* gene. In contrast, in the *B. pseudomallei* σ^E^ mutant, the number of viable bacteria was significantly reduced by the addition of spermidine, indicating the accumulation of spermidine to toxic levels (Figure 3).

This result corroborates the findings from a previous study in *E. coli*, where excess spermidine was shown to be toxic to bacteria and *speG* was shown to be important for bacterial viability [41]. Our study provides further evidence that the regulation of *speG* is affected by σ^E^; *speG* is a novel member of the σ^E^ regulon and σ^E^ plays an indirect but important role in the regulation of polyamine levels in bacterial cells to protect the cells during oxidative stress.

## Conclusions

DNA tiling arrays were employed to identify global transcriptional profile changes in *B. pseudomallei* K96243 exposed to oxidative stress induced by H_2_O_2_. We have identified not only genes involved in repairing cell wall/membrane biosynthesis but also genes involved in energy and metabolism, regulatory and signal transduction, post translational modification, and intracellular trafficking/secretion genes, which are directly regulated by σ^E^ during oxidative stress. We found the increased expression of the *B. pseudomallei* T6SS-4 under oxidative stress. More importantly, we provided evidence that σ^E^ also indirectly regulates the polyamine levels in *B. pseudomallei*, to protect the cells from oxidative stress.

## Methods

### Bacterial strains and culture conditions

*B. pseudomallei* K96243 wild type or the σ^E^ mutant [13] was grown at 37°C in Luria-Bertani (LB) broth or LB agar (Criterion) with or without 50 μg/ml of chloramphenicol (Sigma).

### Extraction of bacterial total RNA

*B. pseudomallei* wild type K96243 or the isogenic σ^E^ mutant was harvested after culturing in LB broth without chloramphenicol supplementation. After centrifugation, the cell pellet was washed and treated with TRIZOL (Invitrogen). One-tenth volume of 1-bromo-3-chloro-propane (Sigma) was added to the mixture before centrifugation. The aqueous phase was transferred to a fresh tube containing equal volume of isopropanol to precipitate the total RNA. After centrifugation, the supernatant was discarded; the RNA pellet was washed with 75% ethanol and suspended in RNase-free water. RNA concentration was quantified by spectrophotometer. The isolated bacterial RNA was then treated with DNase I (Ambion) according to manufacturer’s instructions to remove any genomic DNA contamination. DNase inactivation reagent (Ambion) was then added to stop the reaction.

### Bacterial mRNA enrichment, cDNA synthesis and microarray hybridization

Bacterial mRNA was enriched from purified total RNA and synthesized into single-stranded cDNA prior to microarray hybridization as described in [42]. The purified cDNAs prepared from *B. pseudomallei* wild type or σ^E^ mutant were labeled with Cy5 or Cy3 respectively (Cy5-ULS Cy3-ULS, Kreatech Diagnostics). Hybridization of labeled cDNA to the array was performed and images acquired from array slides as previously described [43]. Data obtained from hybridizations of two independent RNA preparations of each bacterial strain were used in each analysis.

### Design of *B. pseudomallei*K96243 high-density tiling microarray

A high-density tiling array based on the sequenced reference genome *B. pseudomallei* K96243 was custom-fabricated using NimbleGen’s photolithographic Maskless Array Synthesis (MAS) platform (Roche NimbleGen). Using the 7.2 Mb *B. pseudomallei* K96243 genome sequence, we selected 384,926 50 mer oligonucleotide probes to represent both sense and antisense strands of the *B. pseudomallei* genome at an average resolution of 35 bp (probes have a mean overlap of 15 bp). Control features that are not complementary to *B. pseudomallei* K96243 genome, were also included for background checks and alignment purposes. Altogether, 95.1% of the *B. pseudomallei* K96243 genome, including intergenic regions, is represented on this high-density tiling array.

### Data acquisition and preprocessing

Images were acquired with Axon GenePix 4000B laser scanner (Molecular Devices) at 5 μm resolution and intensity data were extracted using the software NimbleScan (Roche NimbleGen). Data obtained from hybridizations of two independent RNA preparations of each sample were used for final analysis. Raw microarray data were first LOWESS (Locally Weighted Scatter Plot Smoother) normalized using GeneSpring GX (Agilent) to correct for dye-bias within array followed by median normalization to normalize across all arrays. Finally, the median ratio of probes corresponding to Sanger’s 5935 genes comparing between *B. pseudomallei* wild type and σ^E^ mutant was computed.

### Differential expression analysis

Changes in the expression of genes under oxidative stress (denoted as T) compared to control conditions (denoted as R) were measured in log_2_ fold change [44]. Specifically, each condition was normalized by a common reference, of which intensity was measured in arrays with Cy5 channels. The common reference (R^c^) is *B. pseudomallei* K96243 grown to stationary phase in LB broth. We computed the difference of two normalized values as the log-transformed fold change: log(*T*/*R*) = log(*T*/*R*^*c*^) − log(*R*/*R*^*c*^). Genes with a log_2_ fold change ≥ 1 were considered further. The microarray data have been deposited in Gene Expression Omnibus (GEO) with the identifier, GSE43205. In particular, the data used in this study are as follow: GSM1058304 (sigmaE-mutant + oxidative stress), GSM1058305 (common reference for sigmaE-mutant + oxidative stress), GSM1058306 (sigmaE-mutant control), GSM1058307 (common reference for sigmaE-mutant control), GSM1058508 (wild type + oxidative stress), GSM1058509 (common reference for wild type + oxidative stress), GSM1058519 (wild type control), GSM1058520 (common reference for wild type control).

### RT-PCR analysis

An overnight cultured of *B. pseudomallei* was sub-cultured in 10 ml of LB broth before incubation at 37°C for 6 h (OD_600_ of 0.8). The logarithmic phase cells were centrifuged and washed with 1x PBS and resuspended into 10 ml of LB broth containing 100 μM H_2_O_2_ before incubation at 37°C for 10 min. After H_2_O_2_ treatment, bacterial RNA was extracted using Total RNA mini Kit (GeneAid) according to the manufacturer’s protocol. To remove trace genomic DNA, the RNA samples were treated with DNase I (Promaga). The yield and purity of the RNA were determined by spectrophotometer (Nanodrop Technologies). The absence of DNA contamination was confirmed by PCR before proceeding to cDNA synthesis.

SuperScript III First-Strand Synthesis System (Invitrogen) was used to convert total RNA to cDNA. The cDNA was amplified using the PCR with primers (Table 2), GoTaq DNA polymerase (Promega) and cycling conditions of 94°C, 3 min and 30 cycles of 94°C for 30 s, 55°C for 1 min, and 72°C for 45 s, followed by incubation at 72°C for 5 min. In each PCR experiment, the amplification of 23S rRNA was used as a normalization control. The amplified products were then visualized using GeneSys software (Syngene). Positive controls were performed with genomic DNA, and negative controls were performed with RNA that had not been subjected to reverse transcription.

**Table 2:** **Oligonucleotide primers used in this study**

Primer sequence (5’ → 3’)		Purpose
23S-F	TTTCCCGCTTAG ATG CTTT	Forward primer for *23S rRNA*
23S-R	AAAGGTACTCTGGGGATAA	Reverse primer for *23S rRNA*
*bpsl0096-*F	TCGATTAGTTCGGCCTCGTG	Forward primer for *bpsl0096*
*bpsl0096-*R	GAGCTCGACTACATCCACCG	Reverse primer for *bpsl0096*
*bpsl0124*-F	ATTATGACGAATGGGAGCAG	Forward primer for *bpsl0124*
*bpsl0124*-R	GCGCTTGTTGATGATGAAAT	Reverse primer for *bpsl0124*
*bpss1434-*F	GTCGAAGGACGTGAACAGTG	Forward primer for *bpss1434*
*bpss1434-*R	ACACGAGAAATTCCGGACAC	Reverse primer for *bpss1434*

### Spermidine sensitivity assay

The numbers of *B. pseudomallei* wild type or the σ^E^ mutant cells were adjusted to 100 CFU and subjected to grow in the presence or absence of 1 mM spermidine (Sigma). After 6 h, the bacterial samples were plated onto LB agar to determine the numbers of viable bacteria as CFU. The cell viability of *B. pseudomallei* under control condition was set as 100%. The viability of *B. pseudomallei* in the presence of spermidine was calculated from CFU count in the presence of spermidine divided by the CFU count of control condition and multiplied by 100.

### Statistical analysis

Average and standard errors of the mean (SEM) were calculated from at least three independent determinations. All tests for significance were performed using the Student’s *t*-test. A *P-*value <0.05 was considered statistically significant.

## Electronic supplementary material

Additional file 1: **Differentially expressed gene of** ***B. pseudomallei*** **K96243 under H**_**2**_ **O**_**2**_ **-induced oxidative stress.** (XLS 120 KB)

Additional file 2: **Differentially expressed gene of** ***B. pseudomallei*** **σ** ^**E**^ **mutant and K96243 wild type under physiological condition.** (XLS 80 KB)

Additional file 3: **Differentially expressed gene of** ***B. pseudomallei*** **σ** ^**E**^ **mutant and K96243 wild type under oxidative stress.** (XLS 130 KB)
